# CeO_2_ Promoted Ni/Al_2_O_3_ Catalyst for the Enhanced Hydrogenolysis of Glucose to 1,2-Propanediol Performance

**DOI:** 10.3390/molecules31030420

**Published:** 2026-01-26

**Authors:** Yu Jiang, Xiaoli Pan, Jifeng Pang, Pengfei Wu, Qinggang Liu, Mingyuan Zheng

**Affiliations:** CAS Key Laboratory of Science and Technology on Applied Catalysis, Dalian Institute of Chemical Physics, Chinese Academy of Sciences, Dalian 116023, China; jiangyu0825@dicp.ac.cn (Y.J.); xlpan@dicp.ac.cn (X.P.); jfpang@dicp.ac.cn (J.P.)

**Keywords:** glucose, 1,2-propanediol, hydrogenolysis, Lewis base site

## Abstract

The selective hydrogenolysis of glucose into 1,2-propanediol (1,2-PG) constitutes a significant yet challenging transformation in biomass valorization, as it involves a highly coupled network of isomerization, C-C bond cleavage, and hydrogenation steps. Herein, a highly efficient Ni-CeO_2_ catalyst supported by basic Al_2_O_3_ is developed via a urea-assisted precipitation strategy. Systematic catalytic evaluation and comprehensive characterization reveal that this synthesis method markedly enhances Ni dispersion and hydrogen activation capacity, while CeO_2_ modification modulates the electronic state of Ni and introduces strong Lewis basic sites associated with oxygen vacancies. The synergistic interplay between Ni and CeO_2_ effectively promotes glucose isomerization and retro-aldol condensation while maintaining sufficient hydrogenation activity. As a result, the optimized catalyst achieves a 1,2-PG yield of 45.1% with over 99% glucose conversion under optimal hydrothermal reaction conditions. Moreover, the catalyst exhibits relatively stable catalytic performance over four consecutive runs. This work elucidates key structure–activity relationships in multifunctional Ni-based catalysts and provides design principles for efficient biomass-derived polyol production.

## 1. Introduction

1,2-Propanediol (1,2-PG) is a fundamental chemical feedstock for synthesizing unsaturated polyester resins [1]. It also serves as a substitute for ethylene glycol, functioning as a base fluid in aircraft deicing formulations and high-quality antifreeze and coolant [2]. Furthermore, owing to its low biotoxicity and environmental compatibility, 1,2-PG is extensively utilized as an effective solvent and humectant across diverse industries, including food, pharmaceuticals, tobacco, and fine chemicals [3]. To date, the industrial production of 1,2-PG heavily relies on the fossil-based propylene oxide liquid-phase hydration process, where propylene oxide and water are mixed in a ratio of 1:15 or greater, with the reaction taking place at 1.8 MPa and 190 °C, grappling with issues including high carbon emissions, high water consumption and energy consumption for separation [4,5]. Consequently, exploring more sustainable routes for 1,2-PG production is of great significance to address the growing market demand and the pressure to reduce carbon emissions.

**Scheme 1:**
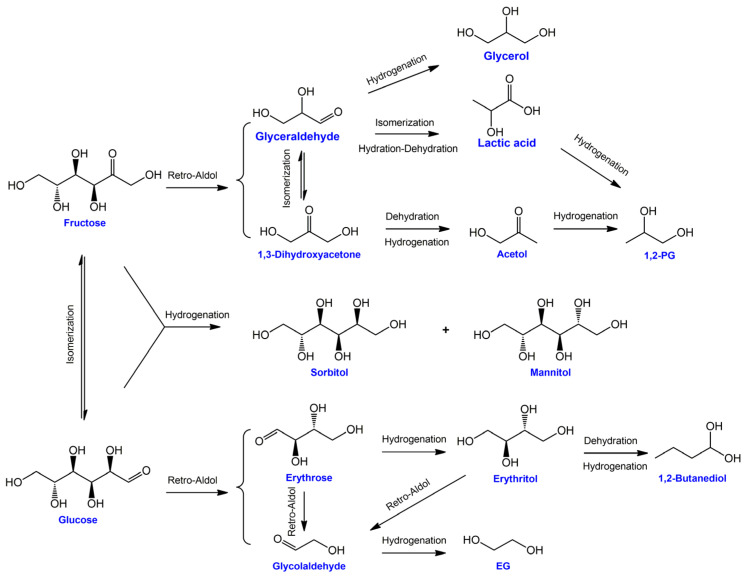
Reaction scheme for conversion of glucose to glycols.

The conversion of hydroxyl-rich biomass derivatives (e.g., cellulose, glucose, fructose, sorbitol, xylitol, glycerol, lactic acid) to produce 1,2-PG offers superior atom economy, as it efficiently reserves the carbon, hydrogen, and oxygen elements present in the feedstock [6,7,8,9,10,11]. Among these, glucose, a low-cost C6 monosaccharide obtained from cellulose and starch, stands out as an ideal raw material for large-scale biorefineries [12]. According to the widely accepted reaction mechanism, the glucose conversion constitutes a complex reaction network involving multiple elementary steps, as shown in Scheme 1 [13]. Briefly, the synthesis of 1,2-PG proceeds through a consecutive pathway: (1) the Lewis acid/base-catalyzed isomerization of glucose to fructose; (2) the retro-aldol condensation of fructose, fragmenting it into key C3 intermediates (e.g., glyceraldehyde, dihydroxyacetone, and lactic acid); and (3) the hydrogenation of these C3 intermediates to yield the desired 1,2-PG. However, it is critical to note that parallel reactions involving dehydration, hydrolysis, and decarbonylation also proceed simultaneously alongside the aforementioned steps. The interconnection of these series and parallel steps means that the relative rates of each reaction dictate the final product distribution. Therefore, the rational design of bifunctional or multifunctional catalysts featuring a balance among distinct active sites is imperative for achieving a high yield of 1,2-PG.

Over the past few decades, considerable research efforts have been performed in developing high-performance catalytic systems, such as Pt-WO_x_/MgO [14], Pt-WO_x_/Al_2_O_3_ [15], Pd-WO_x_/Al_2_O_3_ [16], Ru/C-ZnO [17], Pd-Al/SiO_2_ [18], PtNi/HT [19], Pt/SiO_2_@Mg(OH)_2_ [20], Ni-MgO [21], Ni-MnO_x_/ZnO [22], and Cu-La_2_O_3_/Al_2_O_3_ [23], for glucose to 1,2-PG conversion. The rational design of these catalysts necessitates the coexistence of distinct active sites for hydrogenolysis (facilitated by transition or noble metals), retro-aldol condensation (enabled by inorganic bases or transition metals), and isomerization (catalyzed by metallic components, promoters, or supports). Although inexpensive and readily available nickel-based catalysts are valued for their hydrogenation activity in the hydrothermal conversion of glucose, their widespread application is constrained by an inherent tendency toward excessively strong C-C bond cleavage and insufficient retro-aldol condensation activity [24,25]. To address these limitations and achieve enhanced 1,2-PG selectivity, several strategies are commonly employed, such as co-utilizing inorganic bases (e.g., NaHCO_3_, Ca(OH)_2_), functionalization of the supports, and component modification [26,27,28,29]. Inevitably, such modifications involve trade-offs, potentially complicating product separation, increasing catalyst costs, or compromising catalyst stability.

Herein, a series of Ni-Al_2_O_3_ catalysts were synthesized and evaluated for glucose hydrogenolysis to 1,2-PG. Through systematic screening of supports with varied acid–base characteristics, preparation methods, and various modifying components at different loadings, an optimized Ni_10_Ce_0.5_/Al_2_O_3_-B-UP catalyst was identified and thoroughly investigated. Subsequently, through conditional experiments, this optimized catalyst was demonstrated to achieve a 1,2-PG yield of 45.1% under the optimal reaction condition and maintained relatively stable performance over four consecutive cycles. Finally, the vital physicochemical properties of the catalyst including metal state, texture properties, chemisorption properties, and basicity, were systematically characterized to establish correlations with its catalytic performance.

## 2. Results

### 2.1. Catalytic Conversion of Glucose to 1,2-PG

#### 2.1.1. Catalyst Screening and Optimization

Firstly, a series of Ni/Al_2_O_3_ catalysts with comparable NiO loadings (measured by XRF to be ~10 wt%) were prepared via a conventional impregnation method using three Al_2_O_3_ supports exhibiting distinct acid–base properties. The catalysts were evaluated for glucose hydrogenolysis at 230 °C under 8 MPa hydrogen pressure with a feed rate of 0.5 mL·min^−1^. It should be noted that glucose is intrinsically unstable under these hydrothermal conditions and undergoes degradation or self-polymerization even in the absence of catalytic intervention, resulting in conversions exceeding 99% in all cases. As shown in Table 1 (entries 1–3), none of the three catalysts exhibited satisfactory performance, as reflected by the notably low carbon yields, indicating inefficient catalytic transformation of the substrate.

Among them, the catalyst supported on basic Al_2_O_3_ (Al_2_O_3_-B) afforded a relatively higher 1,2-PG yield of 14.6%, more than twice that obtained over the other supports. Accordingly, Al_2_O_3_-B was identified as the preferred support and subsequently employed for further modification by introducing 0.25 wt% of a second component into the Ni_10_/Al_2_O_3_-B-IM catalyst (Table 1, entries 4–8). Doping with Mn, Mg, or Zn did not lead to a notable improvement in catalytic performance relative to the unmodified Ni_10_/Al_2_O_3_-B-IM catalyst. Although these oxides are known to provide Lewis basic sites that facilitate glucose isomerization, their weak interaction with the support or Ni species likely limits their effectiveness in enhancing 1,2-PG selectivity.

In contrast, modification with rare-earth metals La and Ce resulted in a pronounced enhancement in glucose hydrogenolysis performance. While the yields of ethylene glycol, 1,2-butanediol, and erythritol remained comparable and only a limited amount of glycerol was formed, the yield of the target 1,2-PG increased markedly to 24.2% and 26.1%, respectively. This improvement indicates a preferential enhancement of the reaction pathway leading to 1,2-PG formation. Subsequently, the Ni/Al_2_O_3_-B system was further optimized by screening different catalyst synthesis methods, including co-precipitation (CP) and urea-assisted precipitation (UP). Although similar 1,2-PG yields were obtained over the Ni_10_/Al_2_O_3_-B-CP and Ni_10_/Al_2_O_3_-B-UP catalysts (Table 1, entries 9–10), a pronounced increase in hexitol formation was observed, resulting in a substantial enhancement of the overall carbon yield. This behavior demonstrates the superior hydrogenation activity of catalysts derived from CP and UP methods compared with their impregnation-prepared counterpart.

In pursuit of the dual goal of enhancing catalytic activity and regulating the reaction pathway, the Ni-CeO_2_ binary component combined with the UP synthesis method system was designated for in-depth investigation. As illustrated in Table 1, entries 11–14, the CeO_2_-modified Ni/Al_2_O_3_-B catalysts with varied CeO_2_ doping levels (0.1–1 wt%) were evaluated, and a volcano-shaped dependence was observed between the CeO_2_ loading and the 1,2-PG yield, which reached a maximum of 45.1% at 0.5% CeO_2_ loading. It is hypothesized that at lower CeO_2_ doping levels, the increasing Ce content gradually modified the chemical state of Ni species via the Ni-CeO_2_ interaction. This moderate interaction preserves the catalytic integrity of the primary Ni active sites, as evidenced by the declining hexitol yield alongside a maintained overall carbon yield. Furthermore, serving as a Lewis base, CeO_2_ contributes crucial active sites for steps such as isomerization and retro-aldol condensation. However, excessive CeO_2_ loading predominantly triggers surface coverage effects that compromise the accessibility of Ni active sites. As a comparative reference, the catalyst Ce_0.5_/Al_2_O_3_-B-UP was prepared and evaluated (Table 1, entry 15). The nearly inert activity indicated that Ni served as the primary active component responsible for glucose conversion.

#### 2.1.2. Reaction Condition Influence and Reusability

**Figure 1:**
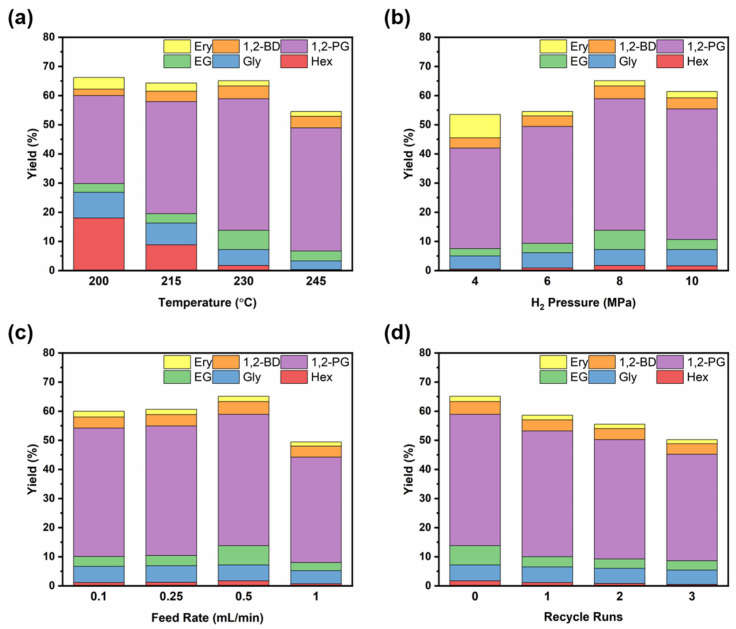
Product yield as a function of (**a**) reaction temperature (p = 8 MPa, V = 0.5 mL/min), (**b**) reaction pressure (T = 230 °C, V = 0.5 mL/min), (**c**) feed rate (p = 8 MPa, T = 230 °C), and (**d**) recycle reaction results (p = 8 MPa, T = 230 °C, V = 0.5 mL/min) over the Ni_10_Ce_0.5_/Al_2_O_3_-B-UP catalyst.

To evaluate the catalytic behavior of the optimized Ni_10_Ce_0.5_/Al_2_O_3_-B-UP catalyst, the reactions were conducted under different conditions. Figure 1a depicts the influence of the reaction temperatures. From 200 °C to 230 °C, the selectivity toward hexitol decreased markedly, while the selectivity for ethylene glycol and 1,2-PG increased, all at a comparable total carbon yield. This shift in product distribution implies that the hydrogenation of C6 sugars proceeds sufficiently even at relatively lower temperatures, while the retro-aldol condensation step becomes the dominant pathway at higher temperatures, consequently promoting the formation of C2 and C3 diols [30]. However, upon raising the reaction temperature to 245 °C, the total yield of liquid products decreased. This decline is attributed to side reactions such as cyclization and partly polycondensation, which promote the transformation of small-molecule intermediates into solid byproducts under such elevated temperatures. The influence of reaction pressure was examined and presented in Figure 1b. In the pressure range of 4 MPa to 8 MPa, a positive correlation was observed between the reaction pressure and the yields of various polyols. This demonstrates that an elevated hydrogen partial pressure enhances the hydrogenation steps involved in the conversion of the substrate and its intermediates. When the pressure was further raised to 10 MPa, no significant change in the product distribution was observed. The yield of 1,2-PG remained largely unchanged at 44.8%, indicating that the reaction system had reached a plateau with respect to pressure under the given conditions. Figure 1c illustrates the influence of the glucose feed rate on the catalytic performance. A relatively stable yield of diols was obtained within the lower feed rate range (0.1–0.25 mL/min). A pronounced decrease in the carbon yield of the liquid products was evident when the feed rate was raised to 1 mL/min. This decrease is consistent with reduced substrate–catalyst contact, implying that the shorter residence time at high feed rates promoted side reactions such as substrate self-polymerization and carbon deposition (coking), thereby reducing the yield of target products.

The catalytic stability was assessed through successive recycling experiments, wherein the catalyst was merely washed with deionized water before each reuse, deliberately omitting any reactivation process. Throughout four operational cycles (Figure 1d), the 1,2-PG yield decreased modestly from 45.1% to 36.6%, accompanied by a decline in carbon yield of liquid products from 65.1% to 50.2%. These declines are presumably attributable to the coverage of active sites by organic deposits. Nevertheless, the negligible leaching of metallic species from the spent catalyst (ICP results: Ni, 9.4%; Ce, 0.46%) underscores its pronounced resistance to structural degradation.

### 2.2. Catalyst Characterization

#### 2.2.1. Crystalline and Textural Properties

**Figure 2:**
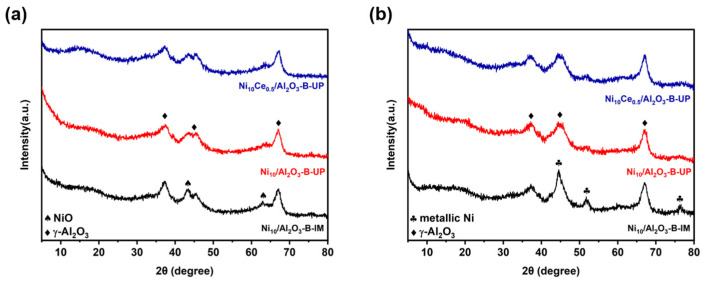
XRD patterns of the (**a**) calcined and (**b**) reduced catalysts.

Three representative catalysts, Ni_10_/Al_2_O_3_-B-IM, Ni_10_/Al_2_O_3_-B-UP, and Ni_10_Ce_0.5_/Al_2_O_3_-B-UP, were designated to characterize their key physicochemical properties and to investigate the corresponding structure-activity relationships. Figure 2 presents the XRD patterns of the calcined and reduced catalysts, respectively. All the samples showed typical broadened diffraction peaks of γ-Al_2_O_3_ at 37.5, 45.8 and 67.0 (2θ degree) [31]. For the calcined Ni_10_/Al_2_O_3_-B-IM, the diffraction peaks at 43.2 and 62.9 (2θ degree) assignable to large NiO particles were observed [31]. In contrast, no diffraction peaks owing to NiO were detected in the catalysts prepared by the UP method, indicating a high dispersion of Ni species. After H_2_ reduction, the XRD pattern of the Ni_10_/Al_2_O_3_-B-IM showed diffraction peaks at 2θ degree of 44.5 (overlapped with γ-Al_2_O_3_), 51.8 and 76.4, which are assigned to the (111), (200) and (220) planes of metallic Ni, respectively [31]. Based on the Scherrer equation, the average Ni crystallite size was determined to be 28.4 nm. Consistent with expectations, the absence of pronounced metallic nickel signals in the UP-synthesized catalysts, with or without Ce modification, suggests that the highly dispersed Ni species resisted aggregation and maintained in high dispersion after reduction. Moreover, the absence of Ce-related signals across all patterns suggested that Ce species were also in a highly dispersed state [32].

**Figure 3:**
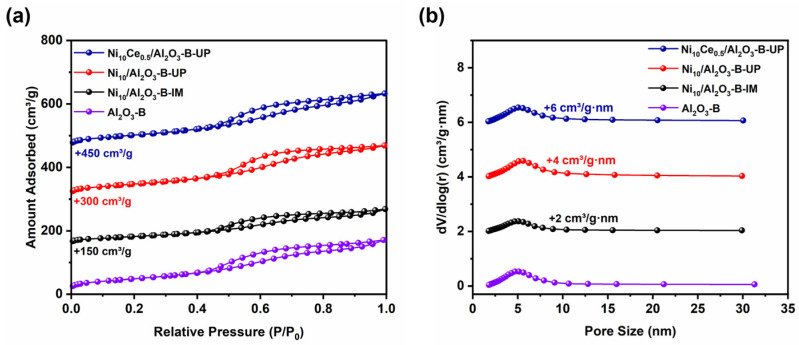
(**a**) N_2_ adsorption–desorption isotherms and (**b**) pore size distribution of samples.

The textural properties of the samples were characterized by N_2_ adsorption–desorption measurements, and the results are presented in Figure 3 and Table 2. All catalysts exhibited a characteristic type IV isotherm with H2 hysteresis, confirming the presence of inherent mesoporous structure. The loading of Ni species via the IM method led to a decrease in the BET surface area (118.0 m^2^/g) and mesopore volume (0.17 cm^3^/g) compared to the bare Al_2_O_3_ support, which is attributed to pore blockage by poorly dispersed Ni species. In contrast, the Ni_10_/Al_2_O_3_-B-UP and Ni_10_Ce_0.5_/Al_2_O_3_-B-UP catalysts showed a significant recovery in BET surface area (172.9 m^2^/g and 188.2 m^2^/g) and a notable increase in mesopore volume (0.27 cm^3^/g and 0.28 cm^3^/g), with the mesopore centered around 4.2 nm and 5.0 nm, respectively. These results indicate that the UP method facilitates the formation of abundant anchoring sites for the metal species, thereby improving their dispersion and preserving the porous texture of the support.

#### 2.2.2. Metal Dispersion and Chemical State

**Figure 4:**
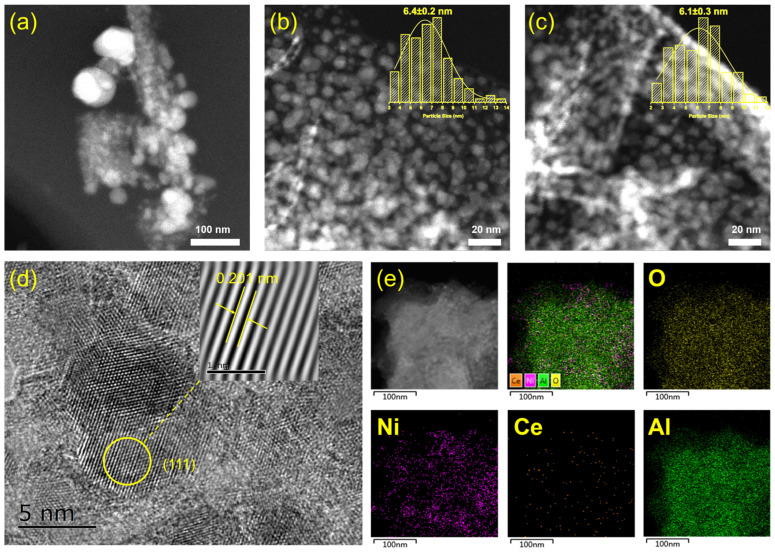
HAADF-STEM images of Ni_10_/Al_2_O_3_-B-IM (**a**), Ni_10_/Al_2_O_3_-B-UP (**b**) and Ni_10_Ce_0.5_/Al_2_O_3_-B-UP (**c**), HRTEM image (**d**) and elemental mapping (**e**) of Ni_10_Ce_0.5_/Al_2_O_3_-B-UP.

The distribution of metal active species on the catalysts was visualized by HAADF-STEM images. As shown in Figure 4a, irregular metallic Ni nanoparticles, with some substantial particles reaching sizes in the tens of micrometers, were observed on the Ni_10_/Al_2_O_3_-B-IM catalyst, indicating the limited capability of the IM method in achieving effective Ni species distribution. In contrast, apparent smaller Ni nanoparticles with an average size of about 6.4 nm and 6.1 nm were relatively uniformly distributed on the Ni_10_/Al_2_O_3_-B-UP and Ni_10_Ce_0.5_/Al_2_O_3_-B-UP catalysts (Figure 4b,c), consistent with the XRD analysis. The comparable average Ni nanoparticle sizes indicates that the favorable dispersion of Ni is primarily governed by the UP synthesis method rather than by the Ce modification. HRTEM analysis of the Ni_10_Ce_0.5_/Al_2_O_3_-B-UP catalyst (Figure 4d) revealed discernible lattice fringes corresponding to metallic nickel crystals (e.g., (111) planes) [33]. However, the characteristic crystallographic planes of Ce species were not detected, likely due to their low abundance and high dispersion, which made clear identification difficult. EDS mapping images (Figure 4e) provided direct evidence that Ce and Ni elements were independently and well-dispersed on the Al_2_O_3_-B support.

**Figure 5:**
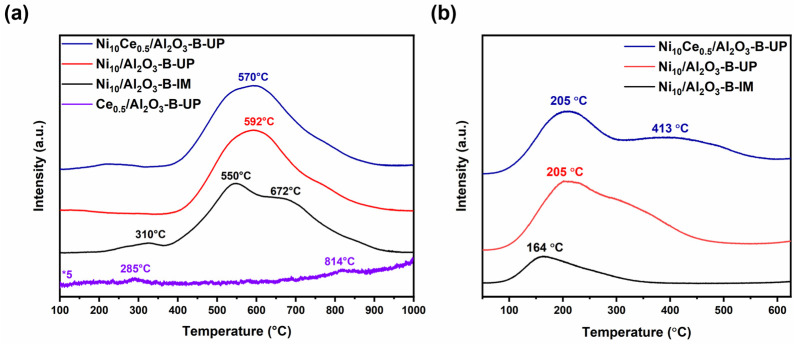
H_2_-TPR (**a**) and H_2_-TPD (**b**) profiles of the catalysts. * the signal was amplified fivefold.

H_2_-TPR experiments were conducted to characterize the reduction behavior of the catalysts. As illustrated in Figure 5a, the profile of the monometallic Ni_10_/Al_2_O_3_-B-IM catalyst exhibited a reduction peak at 310 °C, originating from the reduction in relatively free NiO particles with weak metal–support interactions. A predominant peak appeared at a higher temperature of 550 °C, accompanied by a shoulder at 672 °C, indicating the presence of a small quantity of NiO species that interacts strongly with the support, thus requiring a higher temperature for its reduction [34]. In sharp contrast, the H_2_-TPR profile of the Ni_10_/Al_2_O_3_-B-UP catalyst confirmed a robust metal–support interaction, as demonstrated by the disappearance of the low-temperature peak and the shift in the main reduction peak to a even higher temperature of 592 °C. This variation in interaction strength directly influenced the reducibility of Ni species, which was corroborated by their differing hydrogen consumption (Table 3). The modification with Ce alleviated the metal–support interaction, establishing a Ni-CeO_2_ interaction that resulted in a marginal shift in the reduction peak to a lower temperature (570 °C), thereby enhancing reducibility while maintaining high Ni dispersion [26,35]. For the reference sample Ce_0.5_/Al_2_O_3_-B-UP, the reduction profile was characterized by minimal hydrogen consumption (the signal was amplified fivefold), featuring the partial reduction of CeO_2_ to Ce_2_O_3_ around 285 °C and a minor reduction in trace CeAlO_3_ spinel above 800 °C [36]. This negligible consumption ensured that the reduction profile of the Ni species remained unambiguous and could be analyzed without interference.

Then, H_2_-TPD was employed to identify the surface sites for H_2_ absorption. As shown in Figure 5b, a weak signal observed at 164 °C for the Ni_10_/Al_2_O_3_-B-IM catalyst can be assigned to the desorption of chemisorbed hydrogen from the surface of large Ni particles. This peak shifts to a higher temperature of 205 °C for the UP-derived catalysts, indicating that the UP method strengthens the metal–support interaction and leads to stronger hydrogen binding. Furthermore, the Ni_10_Ce_0.5_/Al_2_O_3_-B-UP catalyst exhibited a new weak peak at 413 °C, possibly originating from hydrogen species bounded strongly on the interfacial CeO_x_ surface in the form of either cerium hydride (Ce-H) species or the spilled-over hydrogen on Ce^3+^ atoms [37]. As shown in Table 3, the quantitative integration of the profiles clearly demonstrated that the UP-derived catalysts achieved a more than three-fold increase in the density of hydrogen adsorption sites compared to the Ni_10_/Al_2_O_3_-B-IM catalyst, which is conducive to the efficacy of hydrogen-related reactions. Moreover, the binary Ni_10_Ce_0.5_/Al_2_O_3_-B-UP catalyst still retained a favorable H_2_ uptakes, attributable to the low concentration of Ce and the effective independent dispersion of both Ni and Ce components.

**Figure 6:**
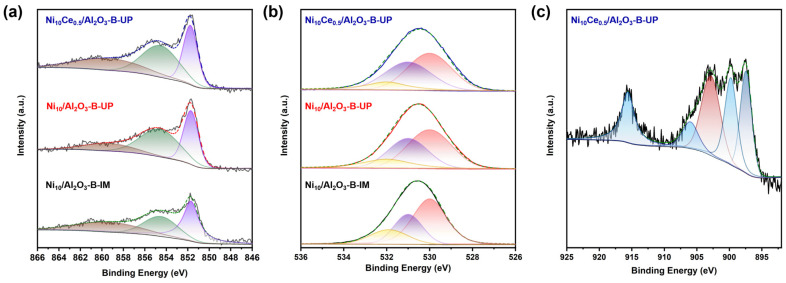
Ni 2p_3/2_ (**a**), O 1s (**b**) and Ce 3d_5/2_ (**c**) XPS of catalysts.

To determine the chemical state of the Ni, O, and Ce species, XPS measurements were conducted on the reduced catalysts. To avoid interference from the overlapping Ni 2p_1/2_ and Ce 3d_3/2_ peaks (860–895 eV), the quantitative analysis was therefore performed on the well-resolved Ni 2p_3/2_ and Ce 3d_5/2_ signals, notwithstanding that the Ce 3d_5/2_ region itself contains a contribution from the Ce 3d_3/2_ signal of Ce^4+^ ions [38]. In the Ni 2p_3/2_ spectra (Figure 6a), two deconvoluted peaks at about 854.5 eV, and 851.7 eV were assigned to the Ni^2+^ and reduced Ni^0^ species, respectively. Satellite peaks of Ni^2+^ were also observed at around 859.3 eV [37]. The relative contents of Ni^0^ and Ni^2+^ in the catalysts are calculated according to the normalized area, and the results are summarized in Table 4. Although the surface of Ni particles may be partially re-oxidized under the ambient condition for the ex situ XPS measurements, the comparative trend in Ni reducibility across different catalysts remains valid under consistent experimental procedures. Accordingly, the metallic Ni and Ni^2+^ species were concomitant on the surface of all catalysts. The lower abundance of metallic Ni (42%) in the Ni_10_/Al_2_O_3_-B-UP catalyst, compared to its IM-prepared counterpart (58%), can be rationalized by the intensified metal–support interaction inherent to the UP synthesis protocol. This stronger interaction stabilizes Ni species in a higher oxidation state, thereby lowering their reducibility. This interpretation is strongly supported by the presented characterization data, as evidenced by the increased Ni dispersion revealed by XRD and TEM, and the decreased H_2_ consumption quantified by H_2_-TPR. With the introduction of Ce, the proportion of metallic Ni and Ni^2+^ was modified to 46% and 54% over the Ni_10_Ce_0.5_/Al_2_O_3_-B-UP catalyst, revealing that CeO_2_ facilitates the reducibility of Ni species, which aligns with the observations from H_2_-TPR. The metal–support interaction was further elucidated by deconvoluting the O 1s spectra (Figure 6b), which revealed three distinct peaks at 530.0 eV (lattice oxygen, O_α_), 531.0 eV (defect sites with low oxygen coordination (oxygen vacancy), O_β_), and 532.0 eV (weakly bound oxygen species, O_γ_, e.g., hydroxyls and adsorbed oxygen). For the monometallic catalyst, the stronger Ni-Al_2_O_3_ interaction constructed by the UP method may lead to the formation of a surface NiAl_2_O_4_ spinel phase. The subsequent reduction process leads to the consumption of surface lattice oxygen and generates structural defects, thereby resulting in a decrease in the proportion of lattice oxygen (48%) and an increase in the proportion of oxygen vacancies (34%). The incorporation of CeO_2_, even in low amounts, significantly enhanced the oxygen vacancy concentration of the catalyst. This enhancement stemmed from CeO_2_’s inherent oxygen storage capacity, which provided numerous oxygen vacancies [35]. Furthermore, hydrogen spillover from metallic Ni sites facilitated the reduction of CeO_2_, inducing additional oxygen vacancy formation.

As illustrated in Figure 6c, the Ce 3d_5/2_ XPS was deconvoluted into characteristic peaks corresponding to Ce^3+^ (902.8 eV) and Ce^4+^ (915.6 eV, 906.0 eV, 899.8 eV), with an additional contribution from the Ce 3d_3/2_ signal of Ce^4+^ (897.4 eV) [36]. Quantitative analysis revealed that the Ce^3+^/(Ce^3+^ + Ce^4+^) ratio reached 35%, which is in good agreement with the highest concentration of oxygen vacancies formed in the Ni_10_Ce_0.5_/Al_2_O_3_-B-UP catalyst. It should be supplemented that the significant presence of Ce^3+^ might also be derived from the redox reaction between Ni^0^ and Ce^4+^ species at the Ni-CeO_2_ interface.

#### 2.2.3. Base Property

**Figure 7:**
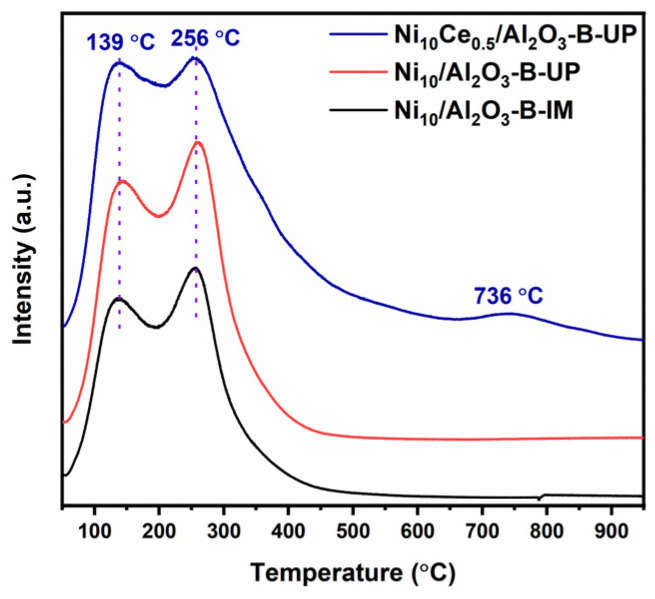
CO_2_-TPD profiles of the catalysts.

CO_2_-TPD technique was employed to characterize the distribution of basic sites on the catalyst surface, as depicted in Figure 7. All catalysts exhibited distinct desorption peaks in the low-temperature region (around 139 °C) and the mid-temperature region (around 256 °C), which are attributed to weak and medium-strength basic sites, respectively. These basic sites primarily originate from the basic alumina support, whose external lattice oxygen and surface hydroxyl groups contribute to the intrinsic moderate basicity. It is noteworthy that a weak desorption peak emerged at a high temperature of 736 °C specifically for the Ni_10_Ce_0.5_/Al_2_O_3_-B-UP catalyst, indicating the presence of very strong basic sites [35,39]. The quantitative data of sites with different basic strengths are summarized in Table 3. The results clearly prove that the introduction of CeO_2_ is exclusively associated with the generation of strong basic sites. This exclusive association can be rationalized by the pronounced tendency of CeO_2_ to form defects. The partial reduction of Ce^4+^ to Ce^3+^ generates oxygen vacancies, which increase the electron density of the adjacent lattice oxygen ions, thereby creating stronger Lewis basic sites [36,40]. Based on these findings, it is postulated that the high yield of 1,2-PG is mediated by these strong basic sites, which are essential for catalyzing the pivotal elementary steps of isomerization and retro-aldol condensation.

### 2.3. Discussion

A series of Ni-Al_2_O_3_ catalysts were applied to the catalytic hydrogenolysis of glucose under hydrothermal conditions for 1,2-PG production. The screening process confirmed an optimal configuration, namely, the combination of a basic alumina support, Ni-Ce binary active components, and the UP synthesis method. A comparative characterization of the physicochemical properties of representative catalysts, coupled with an analysis of the catalytic behavior of the top-performing candidate, enabled the preliminary establishment of the structure-activity relationship within this system, as detailed in the following sections.

The conversion of glucose to 1,2-PG involves a complex network of elementary steps. However, high selectivity toward 1,2-PG in the conversion from glucose is governed by the two key elementary steps: isomerization and retro-aldol condensation. It is well established that glucose isomerization primarily occurs on Lewis acid or base sites. Screening of catalyst supports has confirmed that lattice oxygen in intrinsically basic alumina act as a Lewis base to facilitate this step. In contrast, CeO_2_, characterized by abundant oxygen vacancies, necessitates increased electron density on adjacent lattice oxygen for charge compensation. The resulting strengthened basicity accelerates the crucial first step of glucose-to-1,2-PG conversion while suppressing competing parallel reaction pathways. Furthermore, as a lanthanide catalyst, CeO_2_ exhibits intrinsic catalytic activity for the retro-aldol condensation reaction, thereby synergistically boosting the yield of 1,2-PG.

The geometric and electronic states of the Ni sites, which govern hydrogenation activity, also plays an important role in determining the overall catalytic performance. Characterization techniques (XRD, TEM, BET, H_2_-TPD) unequivocally demonstrated that the UP synthesis method leads to superior Ni dispersion and a higher density of available hydrogenation sites relative to the IM method. This finding explains the poor carbon yield over the IM-prepared monometallic Ni catalyst, attributable to the substrate’s propensity for self-polymerization at the elevated temperature. In contrast, the UP-derived monometallic Ni catalyst exhibited a marked increase in yield of the direct hydrogenation product (hexitols), alongside moderate increases in yields of saturated products like glycerol and diols. Furthermore, while the introduction of CeO_2_ had a minimal impact on Ni dispersion—thus preserving abundant exposed metallic Ni sites—its primary role lies in modulating the electronic state of Ni. This modulation not only appropriately attenuates the metal–support interaction, but also enhances reducibility of Ni species, which is induced by the Ni-CeO_2_ interaction. Consequently, these effects lead to a higher concentration of surface Ni^0^ sites. The unsaturated C2-C4 intermediates generated upstream steps undergo rapid hydrogenation, which leads to an improvement in the overall diol yield.

## 3. Conclusions

In summary, through systematic evaluation of nickel-based catalysts with different supports, modified components, and preparation methods, a high-performance binary Ni-CeO_2_ catalyst supported on a basic alumina was developed via the UP method for glucose hydrogenolysis to 1,2-PG. Under the reaction conditions (230 °C, 8 MPa, and a feed rate of 0.5 mL/min), the optimized catalyst achieved a significantly improved 1,2-PG yield of 45.6%, surpassing the performance of monometallic counterparts prepared by both IM and UP methods. Furthermore, the catalyst demonstrated relatively stable performance over four consecutive cycles, confirming its durability.

Systematic characterizations showed that the UP synthesis strategy effectively promotes the dispersion of Ni species via a strengthened metal–support interaction, thereby generating abundant exposed hydrogenation sites with high activity and markedly improving the overall catalytic performance. Furthermore, Ce modification imparts multiple functionalities, including modulating the electronic state of Ni active species, fine-tuning the metal–support interaction, and providing Lewis basic sites in synergy with the basic alumina support. The synergistic effect between the UP method and Ce modification on the physicochemical properties of the catalyst optimizes the relative rates of the elementary steps within the complex reaction network, resulting in a significant enhancement in 1,2-PG selectivity. This work provides both theoretical and practical guidance for designing multifunctional catalysts for the conversion of biomass to low-carbon diols.

## 4. Materials and Methods

### 4.1. Chemical Reagents

Acidic alumina (Al_2_O_3_-A, 99 wt%), basic alumina (Al_2_O_3_-B, 99 wt%), urea (99 wt%), ammonium carbonate (99.5 wt%), nitric acid (65–68 wt%) and D-glucose (99.5 wt%) were purchased from Shanghai Aladdin Biochemical Technology Co., Ltd. (Shanghai, China). Nano alumina (Al_2_O_3_-N, 99.5 wt%), nickel nitrate hexahydrate (99.5 wt%), cerium nitrate hexahydrate (99.5 wt%), manganese nitrate tetrahydrate (99.5 wt%), magnesium nitrate hexahydrate (99.9 wt%), lanthanum nitrate hexahydrate (99.5 wt%), zinc nitrate hexahydrate (99.9 wt%), ethylene glycol (99 wt%), sorbitol (99 wt%), glycerol (99 wt%), 1,2-propanediol (99 wt%), 1,2-butanediol (99 wt%), and 1,2-pentanediol (99 wt%) were purchased from Shanghai Macklin Biochemical Co., Ltd. (Shanghai, China). All chemicals were used as received without any further purification.

### 4.2. Catalyst Preparation

Incipient wetness impregnation (IM) method: Typically, Ni(NO_3_)_2_·6H_2_O (1.74 g) and Ce(NO_3_)_2_·6H_2_O (0.056 g) were dissolved in H_2_O (3.20 mL). Basic alumina (4.00 g) was added to the solution and stirred fiercely. The product was dried at 100 °C overnight and calcined at 450 °C for 4 h. The obtained sample was denoted as Ni_10_Ce_0.5_/Al_2_O_3_-B-IM, with NiO and CeO_2_ contents of ca. 10 wt% and 0.5 wt%, respectively.

Urea-assisted precipitation (UP) method: Ni(NO_3_)_2_·6H_2_O (1.74 g), Ce(NO_3_)_2_·6H_2_O (0.056 g) and basic alumina (4.00 g) were added in H_2_O (100 mL) and the solution was acidized to pH of 2 by dilute nitric acid. Then, 20 mL of aqueous solution containing 1.15 g urea was added dropwise, stirred at room temperature for 4 h, and at 90 °C for 12 h. The solid product was recovered by filtration and washed with deionized water. Finally, the product was dried at 100 °C overnight and calcined at 400 °C for 4 h. The sample was denoted as Ni_10_Ce_0.5_/Al_2_O_3_-B-UP.

Co-precipitation (CP) method: Ni(NO_3_)_2_·6H_2_O (1.74 g), Ce(NO_3_)_2_·6H_2_O (0.056 g) and basic alumina (4.00 g) were added in H_2_O (100 mL). Then, 15 mL of aqueous solution containing 1.21 g ammonium carbonate was added dropwise until the pH of the mixture reached 8–9. Then, the solid product was recovered by filtration and washed with deionized water. Finally, the product was dried at 100 °C overnight and calcined at 400 °C for 4 h. The sample was denoted as Ni_10_Ce_0.5_/Al_2_O_3_-B-CP.

### 4.3. Characterization Method

Powder X-ray diffraction (XRD) patterns were recorded on a PANalytical X’Pert-Pro X-ray diffractometer (PANalytical B.V., Almelo, The Netherlands) with a Cu K*α* radiation (λ = 0.154059 nm) operating at 40 kV and 40 mA. The chemical composition of the samples was determined using a Philips Magix-601 X-ray fluorescence (XRF) spectrometer (PANalytical B.V., Almelo, The Netherlands). The metal loading of spent catalysts was determined by inductively coupled plasma optical emission spectroscopy (ICP-OES) on a Thermo iCAP6300 instrument (Thermo Fisher Scientific Inc., Waltham, MA, USA). Before analysis, the catalyst was dissolved in aqua regia (3 mL HCl and 1 mL HNO_3_) and then diluted into 1–10 ppm according to the theoretical amount of metals. High-angle annular dark field scanning transmission electron microscopy (HAADF-STEM) images and energy dispersive X-ray spectra (EDX) elemental mapping of samples were obtained by using a JEOL JEM-2100F microscope (Japan Electron Optics Laboratory Co., Ltd., Tokyo, Japan). Temperature-programmed reduction (H_2_-TPR) was carried out on a Micromeritics AutoChem II 2920 instrument (Micromeritics Instrument Co., Norcross, GA, USA) equipped with a thermal conductivity detector (TCD). About 100 mg of calcined catalyst was loaded into a quartz tube and dried in argon stream at 150 °C for 1 h to remove the adsorbed water. After being returned to 50 °C, the catalyst was heated in a 10% H_2_-Ar flow at a heating rate of 10 °C/min up to 800 °C. Ex situ X-ray photoelectron spectra (XPS) were recorded on a Thermo Scientific K-Alpha+ spectrometer (Thermo Fisher Scientific Inc., Waltham, MA, USA) with a monochromatic Al-Kα X-ray source as the excitation source. Prior to the test, the calcined sample was firstly compressed into a thin disk and reduced in a 10% H_2_-Ar flow of 50 mL/min at 600 °C for 4 h and cooled naturally. Then, the pre-reduced sample was passivated in a 1% O_2_-Ar flow of 20 mL/min for 8 h. Afterwards, the sample was carefully transferred into the XPS measurement chamber under high vacuum condition (P < 10^−9^ Pa). Energy corrections were performed using a 1s peak of the polluted carbon at 284.6 eV. Nitrogen adsorption isotherms were measured at −196 °C on a Micromeritics ASAP 2460 system (Micromeritics Instrument Co., Norcross, GA, USA). Before the measurements, all samples were pretreated in vacuum at 300 °C for 8 h. The total surface area was calculated based on the Brunauer–Emmett–Teller (BET) equation. The micropore volume was evaluated using the *t*-plot method. The mesopore volume was evaluated from the adsorption isotherm by the Barrett–Joyner–Halenda (BJH) method. H_2_ temperature-programmed desorption (H_2_-TPD) was performed on Micromeritics Autochem II apparatus (Micromeritics Instrument Co., Norcross, GA, USA) equipped with a mass spectrometer (MS) detector. Typically, 100 mg of sample was pretreated at 550 °C for 120 min in 10% H_2_-Ar flow of 30 mL/min and purged in Ar flow of 30 mL/min for 60 min. After cooling down to 40 °C, H_2_ was dosed onto the sample until its saturated adsorption. Then the sample was purged with He flow for 30 min to remove the gaseous H_2_. Finally, the sample was heated to 800 °C at a rate of 10 °C/min, and the H_2_ desorbed from the surface and was monitored by the MS detector. CO_2_ temperature-programmed desorption (CO_2_-TPD) was performed on Micromeritics Autochem II apparatus equipped with a MS detector. Typically, 100 mg of sample was pretreated at 500 °C for 60 min in He flow of 30 mL/min. After cooling down to 50 °C, CO_2_ was dosed onto the sample until its saturated adsorption. Then the sample was purged with He flow for 30 min to remove the physically absorbed CO_2_. Finally, the sample was heated to 800 °C at a rate of 10 °C/min, and the CO_2_ desorbed from the surface and was monitored by the MS detector.

### 4.4. Catalyst Evaluation

The catalytic conversion of glucose was carried out in a high-pressure batch reactor. Before the catalytic studies, the catalyst was reduced in a flow of H_2_ at 600 °C for 2 h and passivated in a flow of 1% O_2_/N_2_ for 12 h at room temperature. Typically, 1.0 g of catalysts and 20 mL of water were charged in a 100 mL stainless-steel autoclave (Parr Instrument Co., Moline, IL, USA). The reactor was then flushed with 0.2 MPa hydrogen for 5 times and filled with 4 MPa hydrogen. The mixture was stirred at a rate of 800 rpm and heated to 230 °C until the pressure reached 8 MPa. After the reactor reached the target temperature and pressure, 20 mL of 10 wt.% glucose solution was fed into the reactor by a high-pressure pump at a constant rate of 0.5 mL·min^−1^ (feeding time = 40 min). Thereafter, the reaction was maintained for a further 30 min under the same conditions. The total reaction time, including feeding, was 70 min. For stability tests, the catalyst was filtered and rinsed with deionized water for 3 times and then put into the reactor for the next run.

After the reaction, the reactor was cooled down to room temperature, and the liquid products were analyzed by high-performance liquid chromatography (Agilent HPLC 1100, Agilent Technologies Inc., Santa Clara, CA, USA) equipped with a Rezox™ ROA-Qrganic Acid H^+^ column, refractive index detector (cell temperature of 45 °C), and 5 mM sulfuric acid aqueous was used as the mobile phase with 0.5 mL min^−1^ flow rate.

The products yield and glucose conversion were calculated as follows:

$$Yield\left(\%\right)=\frac{mol\;of\;carbon\;in\;the\;target\;products}{mol\;of\;carbon\;in\;the\;substrate\;fed\;into\;the\;reactor}\;\times \;100\%$$

$$Conversion\left(\%\right)=\frac{mol\;of\;carbon\;in\;the\;converted\;substate}{mol\;of\;carbon\;in\;the\;substrate\;fed\;into\;the\;reactor}\times 100\%$$

## Figures and Tables

**Table 1:** Results of the catalytic hydrogenolysis of glucose over various catalysts ^a^.

Ent.	Catalyst	Method	Conv./%	Yields ^b^/%						
				Hex	Gly	EG	1,2-PG	1,2-BD	Ery	Carbon Balance
1	Ni_10_/Al_2_O_3_-A	IM	>99	5.9	9.3	2	6.6	0.4	1.6	25.8
2	Ni_10_/Al_2_O_3_-N	IM	>99	5.6	10.4	6.6	7.3	0.9	1.7	32.5
3	Ni_10_/Al_2_O_3_-B	IM	>99	4.2	0	1.4	14.6	2	1.2	23.4
4	Ni_10_Mn_0.25_/Al_2_O_3_-B	IM	>99	1.8	7.3	5.7	10.4	0	1.2	26.4
5	Ni_10_Mg_0.25_/Al_2_O_3_-B	IM	>99	0	1.2	1.8	14.3	2.5	0.5	20.3
6	Ni_10_Zn_0.25_/Al_2_O_3_-B	IM	>99	0	2.5	1.3	14.7	2.6	0.5	21.6
7	Ni_10_La_0.25_/Al_2_O_3_-B	IM	>99	4.7	8.6	2.8	24.2	2.1	1.0	43.4
8	Ni_10_Ce_0.25_/Al_2_O_3_-B	IM	>99	2.9	4.3	2.4	26.1	2.8	0.8	39.3
9	Ni_10_/Al_2_O_3_-B	CP	>99	14.1	12.2	7.2	12.0	3.0	4.5	53.0
10	Ni_10_/Al_2_O_3_-B	UP	>99	24.5	6.6	1.5	15.5	1.6	8.2	57.9
11	Ni_10_Ce_0.1_/Al_2_O_3_-B	UP	>99	7.2	8.2	6.2	29.3	3.8	2.5	57.2
12	Ni_10_Ce_0.25_/Al_2_O_3_-B	UP	>99	2.0	7.2	4.1	43.6	3.9	2.2	63.0
13	Ni_10_Ce_0.5_/Al_2_O_3_-B	UP	>99	1.7	5.5	6.6	45.1	4.4	1.7	65.1
14	Ni_10_Ce_1.0_/Al_2_O_3_-B	UP	>99	0.5	3.2	2.5	37.1	4.0	1.2	48.5
15	Ce_0.5_/Al_2_O_3_-B	UP	>99	0.2	0.5	1.2	0.5	0.1	0.2	2.7

^a^ p = 8 MPa, T = 230 °C, V = 0.5 mL/min, t = 30 min. ^b^ Hex: sorbitol and mannitol, Gly: glycerol, EG: ethylene glycol, 1,2-PG: 1,2-propanediol, 1,2-BD: 1,2-butanediol, Ery: erythritol.

**Table 2:** Composition and textural properties of the catalysts.

Catalyst	Composition (%) ^a^		S_BET_ (m^2^/g) ^b^	V_micro_ (cm^3^/g) ^c^	V_meso_ (cm^3^/g) ^d^	Pore Size (nm) ^e^
	NiO	CeO_2_				
Ni_10_Ce_0.5_/Al_2_O_3_-B-UP	10.11	0.51	188.2	0	0.28	5.0
Ni_10_/Al_2_O_3_-B-UP	10.05	-	172.9	0	0.27	4.2
Ni_10_/Al_2_O_3_-B-IM	10.13	-	118.0	0	0.17	4.6
Al_2_O_3_-B	-	-	179.6	0	0.27	4.7

^a^ Determined by XRF. ^b^ BET surface area. ^c^ t-Plot micropore volume. ^d^ BJH adsorption volume. ^e^ BJH mesopore size distribution based on the adsorption branch.

**Table 3:** Ni particle sizes, reduction and chemisorption properties of the catalysts.

Catalyst	Ni Particle Size ^a^ (nm)	Ni Particle Size ^b^ (nm)	Consumption of H_2_^c^ (mmol/g)	H_2_ Uptake ^d^ (mmol/g)	CO_2_ Uptake ^e^ (mmol/g)	
					Low and Medium Temperature	High Temperature
Ni_10_Ce_0.5_/Al_2_O_3_-B-UP	-	6.1	1.72	0.104	0.48	0.10
Ni_10_/Al_2_O_3_-B-UP	-	6.4	1.70	0.109	0.46	-
Ni_10_/Al_2_O_3_-B-IM	28.4	10–60+	1.84	0.030	0.32	-

^a^ Calculated by the Scherrer equation at 2θ = 51.4°. ^b^ Obtained from the HAADF-STEM images. ^c^ Determined by H_2_-TPR. ^d^ Determined by H_2_-TPD. ^e^ Determined by CO_2_-TPD.

**Table 4:** Types and contents of elements in catalysts.

Catalyst	Contents of Ni Species ^a^ (%)		Contents of O Species ^b^ (%)			Contents of Ce Species ^c^ (%)	
	Ni^2+^	Ni^0^	O_α_	O_β_	O_γ_	Ce^4+^	Ce^3+^
Ni_10_Ce_0.5_/Al_2_O_3_-B-UP	54	46	47	39	14	65	35
Ni_10_/Al_2_O_3_-B-UP	58	42	48	34	18	-	-
Ni_10_/Al_2_O_3_-B-IM	42	58	55	27	17	-	-

^a^ Determined by Ni 2p_3/2_ spectra. ^b^ Determined by O 1s spectra. ^c^ Determined by Ce 3d_5/2_ spectra.

## Data Availability

The original contributions presented in this study are included in the article. Further inquiries can be directed to the corresponding authors.
